# HDAC8 functions in spindle assembly during mouse oocyte meiosis

**DOI:** 10.18632/oncotarget.15383

**Published:** 2017-02-16

**Authors:** Kemei Zhang, Yajuan Lu, Chaohua Jiang, Wei Liu, Jing Shu, Xueqin Chen, Yingjiao Shi, Ensheng Wang, Li Wang, Qinbo Hu, Yibo Dai, Bo Xiong

**Affiliations:** ^1^ Reproductive Medicine Center, Ningbo First Hospital, Zhejiang 315010, China; ^2^ College of Animal Science and Technology, Nanjing Agricultural University, Nanjing 210095, China

**Keywords:** HDAC8, spindle assembly, chromosome alignment, kinetochore-microtubule attachment, aneuploid eggs

## Abstract

HDAC8 is a class I histone deacetylase that functions in a variety of biological processes through its non-histone substrates. However, its roles during oocyte meiosis remain elusive. Here, we document that HDAC8 localizes at spindle poles and positively participates in the regulation of microtubule organization and spindle assembly in mouse oocytes. Depletion of HDAC8 by siRNA-based gene silencing results in various spindle defects and chromosome misalignment during oocyte meiotic maturation, accompanied by impaired kinetochore-microtubule attachments. Consequently, a higher incidence of aneuploidy is generated in HDAC8-depleted MII eggs. In addition, inhibition of HDAC8 activity with its selective inhibitor PCI-34051 phenocopies the spindle/chromosome defects resulting from HDAC8 depletion by siRNA injection. Finally, we find that HDAC8 is required for the correct localization of ϕ-tubulin to spindle poles. Collectively, these data reveal that HDAC8 plays a significant role in regulating spindle assembly and thus ensuring the euploidy in mouse eggs.

## INTRODUCTION

In mammals, meiosis is a specialized type of cell division that reduces the chromosome by half, resulting in haploid cells. This cell division is divided into meiosis I and meiosis II. Meiosis I is a process that homologous chromosomes are segregated and joined as tetrads, producing two haploid cells. And meiosis II is the second meiotic division involving the separation of sister chromatids [1, 2]. Errors in these processes, including deficient structure of spindle and unbalanced allocation of chromosomes could lead to the generation of aneuploidy [3, 4]. And it is widely believed that an extra or missing chromosome is the most frequent cause of genetic disorders as well as cancers [5, 6]. In addition, almost one-third of meiosis II errors were associated with the preceding meiosis I errors [7]. Thus, it is of significant importance to regulate and monitor the accurate segregation of chromosomes, ensuring the right distribution of genetic material in each daughter cell [8].

Histone deacetylase (HDACs) is a family of enzymes that remove acetyl groups from an ε-N-acetyl lysine amino acid on a histone, facilitating the combination of histones and DNA, thus causing global chromatin condensation and transcription repression [9, 10]. To data, there are 18 HDAC proteins that have been identified and grouped into four classes based on their sequence homology to yeast proteins [11]. Class I contains HDAC1, HDAC2, HDAC3 and HDAC8. Class II includes HDAC4, HDAC5, HDAC6, HDAC7, HDAC9 and HDAC10. SIRTs 1, 2, 3, 4, 5, 6, and 7 belong to class III, and HDAC11 represents class IV [12–14]. It has long been thought that HDACs are responsible for deacetylation of nucleosomal histones solely. However, accumulating recent studies have demonstrated that HDACs actually have broader biological functions besides the regulation of gene transcription within the cells. They have been implicated in many critical signaling networks that are linked to a variety of diseases, such as cancers, neurodegenerative disorders and metabolic dysregulation [15–17].

HDAC8, a predominantly cytoplasmic deacetylase, has been found to have various non-histone substrates to function in different biological events. It is colocalized with α-SMA to control the smooth muscle contractility [18–20]. Mutation of HDAC8 results in increased SMC3 acetylation level and inefficient dissolution of cohesin complex released from chromatin in prophase and anaphase during the cell cycle, leading to Cornelia de Lange syndrome (CdLS) [21, 22]. Knockdown of HDAC8 by RNA interference inhibits the proliferation of lung, colon, and cervical cancer cell lines, whereas up-regulation of HDAC8 promotes the proliferation and inhibits apoptosis in hepatocellular carcinoma [15, 23]. In parasitic and viral infection, HDAC8 is required for centrosome cohesion and virus entry, and down-regulation of HDAC8 expression in schistosomula results in a decreased capability of their survival and maturation in infected mice [24, 25].

In this report, we aim to investigate the potential role of HDAC8 during mouse oocyte meiotic maturation. Our findings reveal that HDAC8 localizes at spindle poles to participate in proper spindle assembly and chromosome alignment, protecting mouse eggs from aneuploidy. Additionally, the involvement of HDAC8 in spindle assembly is mediated by γ-tubulin.

## RESULTS

### HDAC8 localizes at the spindle poles during mouse oocyte meiosis

**Figure 1:**
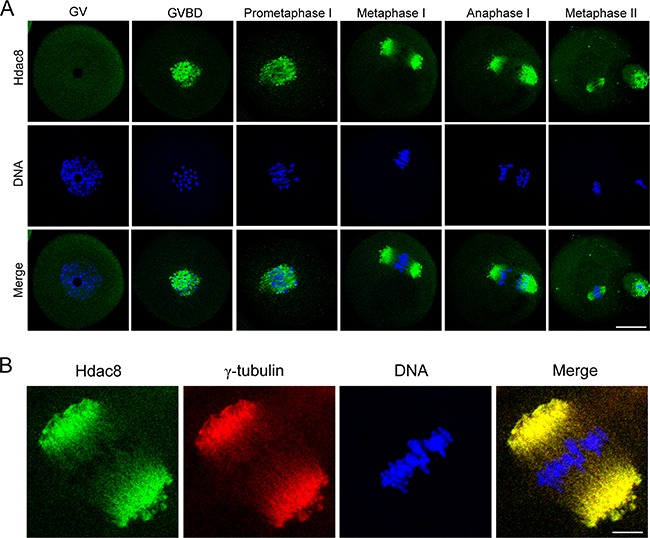
Subcellular localization of HDAC8 during mouse meiotic maturation **A**. Mouse oocytes at GV, prometaphase I, metaphase I, anaphase I and metaphase II stages were immunolabeled with anti- HDAC8 antibody (green) and counterstained with Hoechst (blue). Images were acquired under the confocal microscope. Scale bar, 20 μm. **B**. Metaphase I oocytes were double-stained with anti-HDAC8 antibody (green) and anti-γ-tubulin antibody (red) and then counterstained with Hoechst (blue). Scale bar, 5 μm.

We firstly examined the subcellular localization of HDAC8 from GV (Germinal Vesicle) to MII (Metaphase II) stages by immunofluorescent analysis. We found that HDAC8 widely distributed in the cytoplasm of mouse oocytes at GV stage (Figure 1A). Shortly after GVBD (Germinal Vesicle Breakdown) stage, HDAC8 started to accumulate around the chromosomes (Figure 1A). Strikingly, HDAC8 showed a spindle pole-like localization pattern in both MI (Metaphase I) and M II (Metaphase II) stages of mouse oocytes (Figure 1A). To further confirm the localization relationship between HDAC8 and spindle apparatus, we double stained HDAC8 with the spindle pole protein γ-tubulin, a member of tubulin family, which is found initially in the MTOCs (microtubule organizing centers) and spindle poles and has a significant role in the polar orientation of microtubules. As expected, the results showed that the fluorescent signals of HDAC8 and γ-tubulin were clearly overlapped in MI oocytes (Figure 1B), demonstrating that HDAC8 localizes at the spindle poles during mouse oocyte meiotic maturation.

### HDAC8 maintains normal spindle assembly and chromosome alignment in mouse oocytes

**Figure 2:**
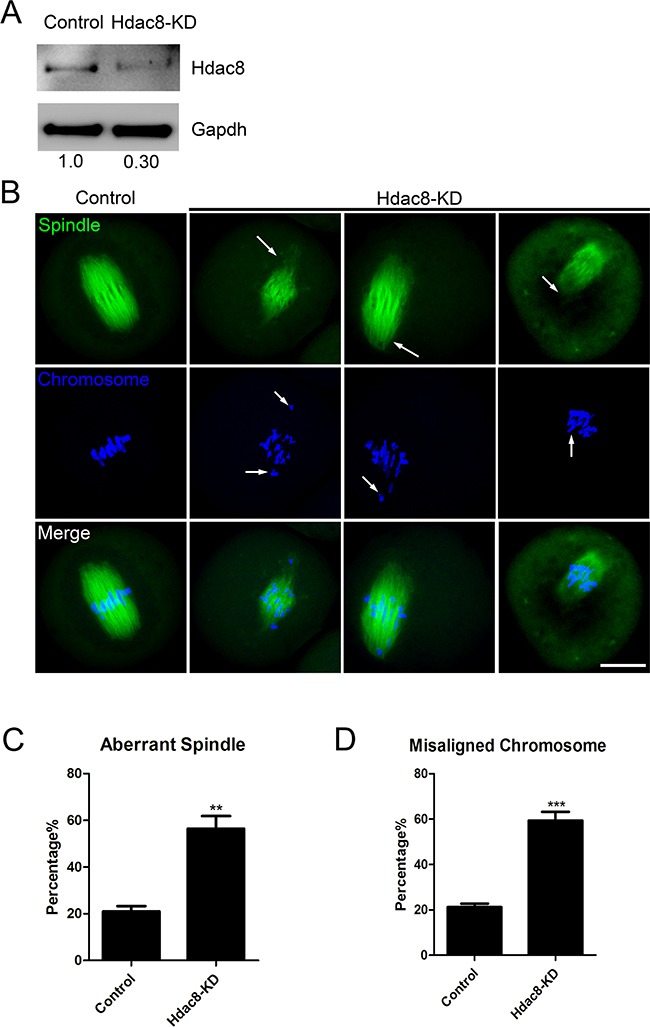
Depletion of HDAC8 causes spindle and chromosome abnormalities **A**. Protein levels of HDAC8 in control and HDAC8-KD (siRNA microinjected) oocytes. The blots were probed with anti-HDAC8 antibody and anti-Gapdh antibody, respectively. **B**. Representative images of spindle morphologies and chromosome alignment in control and HDAC8-KD oocytes. Oocytes were immunostained with anti-α-tubulin-FITC antibody to visualize spindles and counterstained with Hoechst to visualize chromosomes. Scale bar, 20μm. **C**. The rate of aberrant spindles was recorded in control and HDAC8-KD oocytes. Data were presented as mean percentage (mean ± SEM) of at least three independent experiments. Asterisk denotes statistical difference at a *p < 0.05* level of significance. **D**. The rate of misaligned chromosomes was recorded in control and HDAC8-KD oocytes. Data were presented as mean percentage (mean ± SEM) of at least three independent experiments. Asterisk denotes statistical difference at a *p < 0.05* level of significance.

To define the role of HDAC8 during oocyte meiosis, loss-of-function experiments through gene-targeting siRNA microinjection were employed to deplete HDAC8. Knockdown effect was tested by western blotting analysis. The result revealed that HDAC8 protein level was significantly reduced in HDAC8-depleted oocytes compared to controls (Figure 2A), indicating that HDAC8 was successfully depleted by RNA interference. The spindle pole localization prompted us to propose that HDAC8 might be involved in spindle assembly. To test this, M I oocytes were immunostained with anti-α-tubulin antibody to display spindle morphology and counterstained Hoechst to show the chromosomes. We found a variety of aberrant disorganized spindle apparatus with misalignment chromosomes after HDAC8 depletion (Figure 2B). The quantification result showed that a higher percentage of defective spindle/chromosome abnormalities was observed in HDAC8-depleted oocytes than that in controls (Spindle: 56.45 ± 5.39%, n=99 vs 21.04 ± 2.26%, n=99 control, *p* < 0.05; Chromosome: 59.37 ± 3.89%, n=99 vs 21.23 ± 1.52%, n=99 control, *p* < 0.05; Figure 2C, 2D), indicating that HDAC8 plays a considerable role in spindle assembly and chromosome alignment in mouse oocytes.

### HDAC8 is required for correct kinetochore-microtubule attachments to maintain euploidy in mouse oocytes

**Figure 3:**
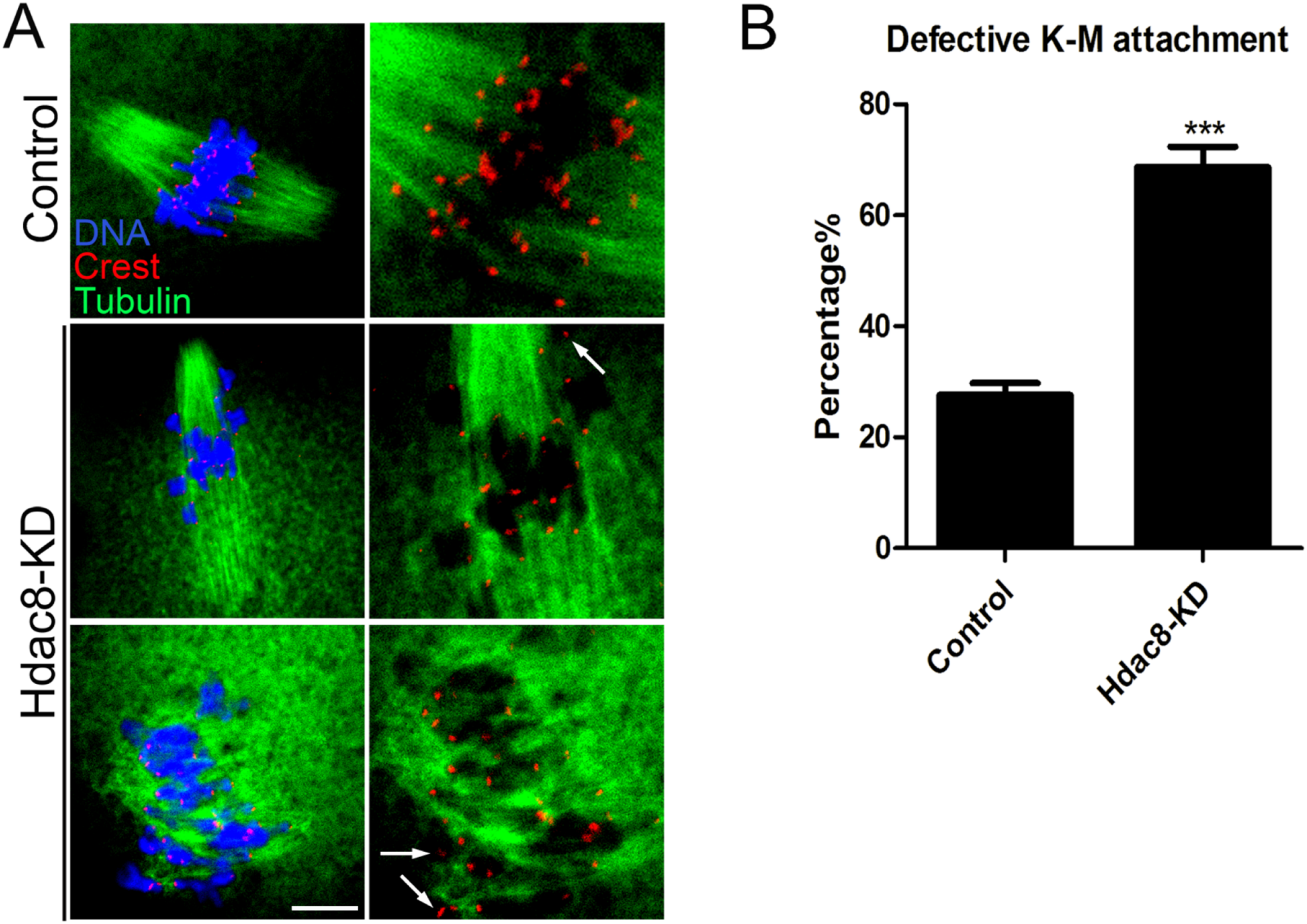
Depletion of HDAC8 compromises K-M attachments **A**. Representative images of K-M attachments in control and HDAC8-KD oocytes. Oocytes were immunostained with anti-α-tubulin-FITC antibody to visualize spindles, with CREST to visualize kinetochores, and counterstained with Hoechst to visualize chromosomes. Scale bar, 5 μm. **B**. The rate of defective K-M attachments was recorded in control and HDAC8-KD oocytes. Data were presented as mean percentage (mean ± SEM) of at least three independent experiments. Asterisk denotes statistical difference at a *p < 0.05* level of significance.

Kinetochore is a unique protein complex on the centromere where the spindle microtubules attach during the cell division to segregate sister chromatids. The remarkably higher ratio of spindle/chromosome defects in HDAC8-depleted oocytes predicted the generation of unattached kinetochore-microtubule. To clearly visualize the interaction between kinetochores and microtubules, mouse oocytes were exposed to cold treatment to depolymerize the unstable microtubules which are not attached to kinetochores, followed by immunostaining with α-tubulin and CREST antibodies. Indeed, we observed, via confocal scanning microscope, that HDAC8 depletion caused a substantially elevated frequency of scattered kinetochores with rare cold-stable microtubules attached (Figure 3A). By performing quantitative analysis, the data showed that over 60% of impaired K-M attachments was present in HDAC8-depleted oocytes compared to around 20% in controls (68.70 ± 3.66%, n=68 vs 27.64 ± 2.12%, n=69 control, *p* < 0.05; Figure 3B). These insufficient attachments of kinetochore-microtubule would possibly lead to the unstable chromosome biorientation, which might largely be associated with the generation of aneuploidy.

**Figure 4:**
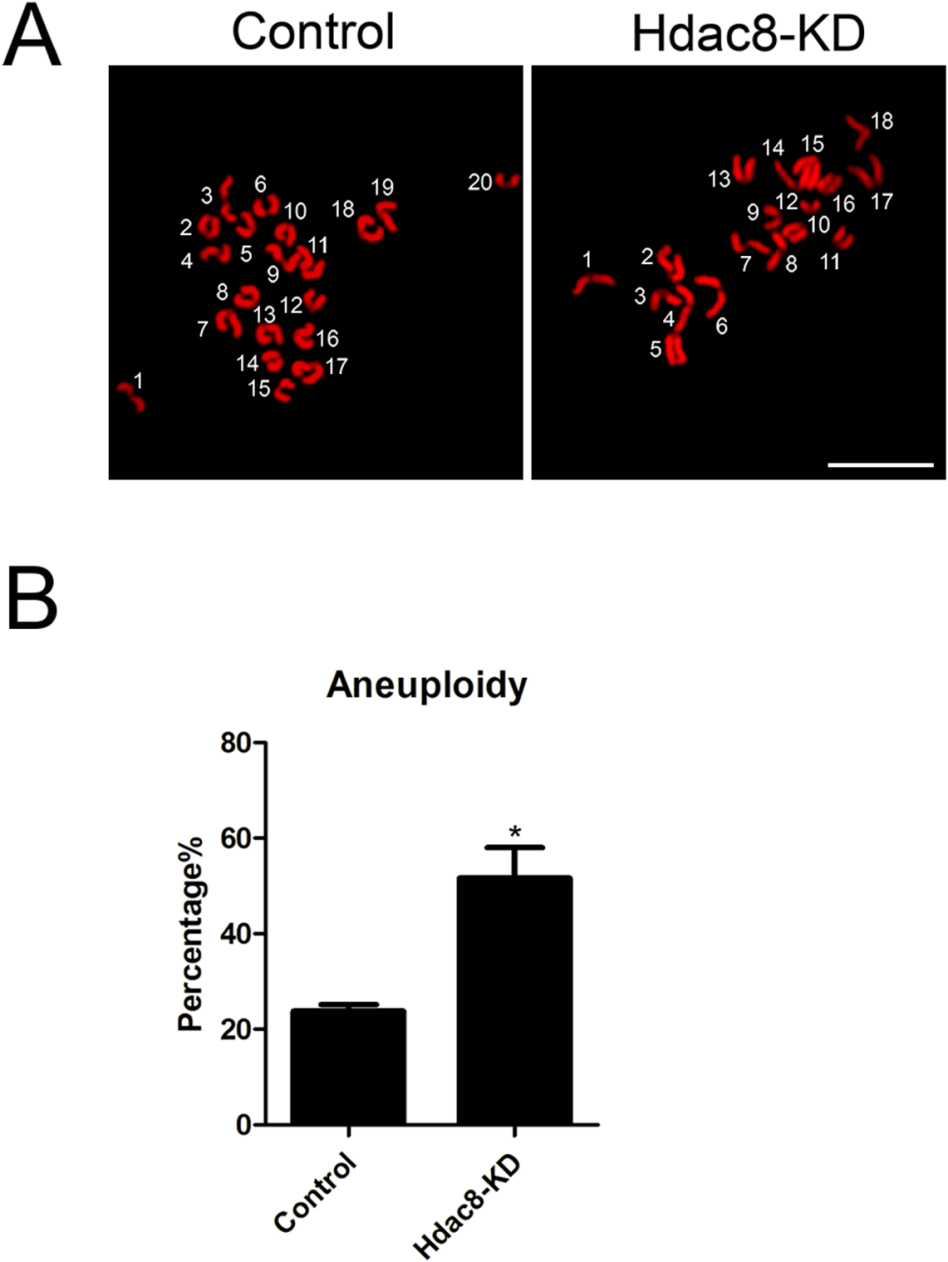
Depletion of HDAC8 generates aneuploid eggs **A**. Representative images of euploid and aneuploid MII eggs. Chromosome spreading was performed to count the number of chromosomes. Chromosomes were counterstained with PI. Scale bar, 2.5 μm. **B**. The rate of aneuploidy was recorded in control and HDAC8-KD eggs. Data were presented as mean percentage (mean ± SEM) of at least three independent experiments. Asterisk denotes statistical difference at a *p < 0.05* level of significance.

Thus, we next tested the incidence of aneuploidy following HDAC8 depletion. To this end, we carried out the karyotypic analysis of MII eggs by chromosome spreading. As shown in Figure 4A, control oocytes owned a correct number of 20 univalents to maintain the euploidy. The HDAC8-depleted oocytes, however, showed a more or less than 20 univalents, whose defective frequency was markedly higher than that of controls (51.65 ± 6.38%, n=45 vs 23.76 ± 1.42%, n=50 control, *p* < 0.05; Figure 4B). All together, these results imply that depletion of HDAC8 was unsuspiciously correlated to the impairment of spindle/chromosome structure as well as K-M attachments, resultantly leading to a higher occurrence of aneuploid eggs.

### Deacetylase activity of HDAC8 is indispensable for its functions in spindle assembly and maintenance of euploidy in mouse oocytes

**Figure 5:**
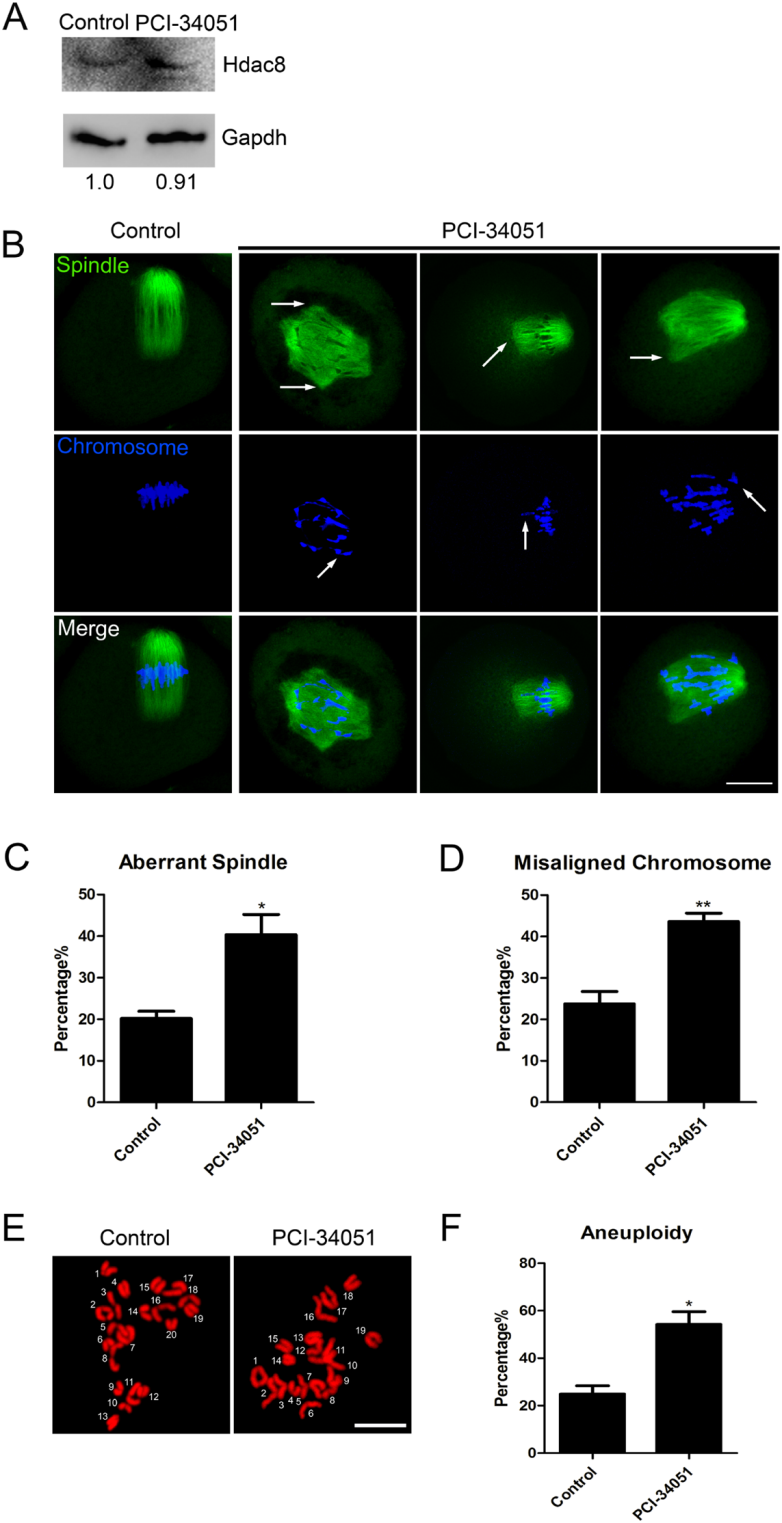
Inhibition of HDAC8 enzymatic activity causes spindle/chromosome abnormalities and generates aneuploid eggs **A**. Protein levels of HDAC8 in control and PCI-34051-treated oocytes. The blots were probed with anti-HDAC8 antibody and anti-Gapdh antibody, respectively. **B**. Representative images of spindle morphologies and chromosome alignment in control and PCI-34051-treated oocytes. Oocytes were immunostained with anti-α-tubulin-FITC antibody to visualize spindles and counterstained with Hoechst to visualize chromosomes. Scale bar, 20μm. **C**. The rate of aberrant spindles was recorded in control and PCI-34051-treated oocytes. Data were presented as mean percentage (mean ± SEM) of at least three independent experiments. Asterisk denotes statistical difference at a *p < 0.05* level of significance. **D**. The rate of misaligned chromosomes was recorded in control and PCI-34051-treated oocytes. Data were presented as mean percentage (mean ± SEM) of at least three independent experiments. Asterisk denotes statistical difference at a *p < 0.05* level of significance. **E**. Representative images of euploid and aneuploid MII eggs. Chromosome spreading was performed to count the number of chromosomes. Chromosomes were counterstained with PI. Scale bar, 2.5 μm. **F**. The rate of aneuploid eggs was recorded in control and PCI-34051-treated oocytes. Data were presented as mean percentage (mean ± SEM) of at least three independent experiments. Asterisk denotes statistical difference at a *p < 0.05* level of significance.

Another strategy to investigate the function of HDAC8 is to inhibit its enzymatic activity. For this purpose, oocytes were cultured in the medium supplemented with HDAC8-selective inhibitor PCI-34051. Western blotting result showed that inhibition of HDAC8 enzymatic activity did not have effects on its protein level (Figure 5A). Then, we asked whether the spindle formation and chromosome alignment would suffer from damage as a result of HDAC8 enzymatic inhibition. To gain insights into this question, immunostaining was performed to visualize the spindle and chromosome in control and HDAC8-inhibited oocytes. As expected, a higher rate of spindle malformation and chromosome alignment failure was present in the HDAC8-inhibited oocytes compared to controls (Spindle: 40.35 ± 4.87%, n=96 vs 20.17 ± 1.76%, n=94 control, *p* < 0.05; Chromosome: 43.60 ± 2.04%, n=96 vs 23.75 ± 2.98%, n=94 control, *p* < 0.05; Figure 5B, 5C, 5D). Also, Inhibition of HDAC8 significantly increased the incidence of aneuploidy rate from about 20% in controls to over 50% (54.21 ± 5.44%, n=31 vs 24.89 ± 3.46%, n=47 control, *p* < 0.05; Figure 5E, 5F). Collectively, these observations indicate that the regulation of HDAC8 in spindle assembly and maintenance of euploidy is dependent on its deacetylase activity.

### Loss of HDAC8 disrupts the localization γ-tubulin in mouse oocytes

**Figure 6:**
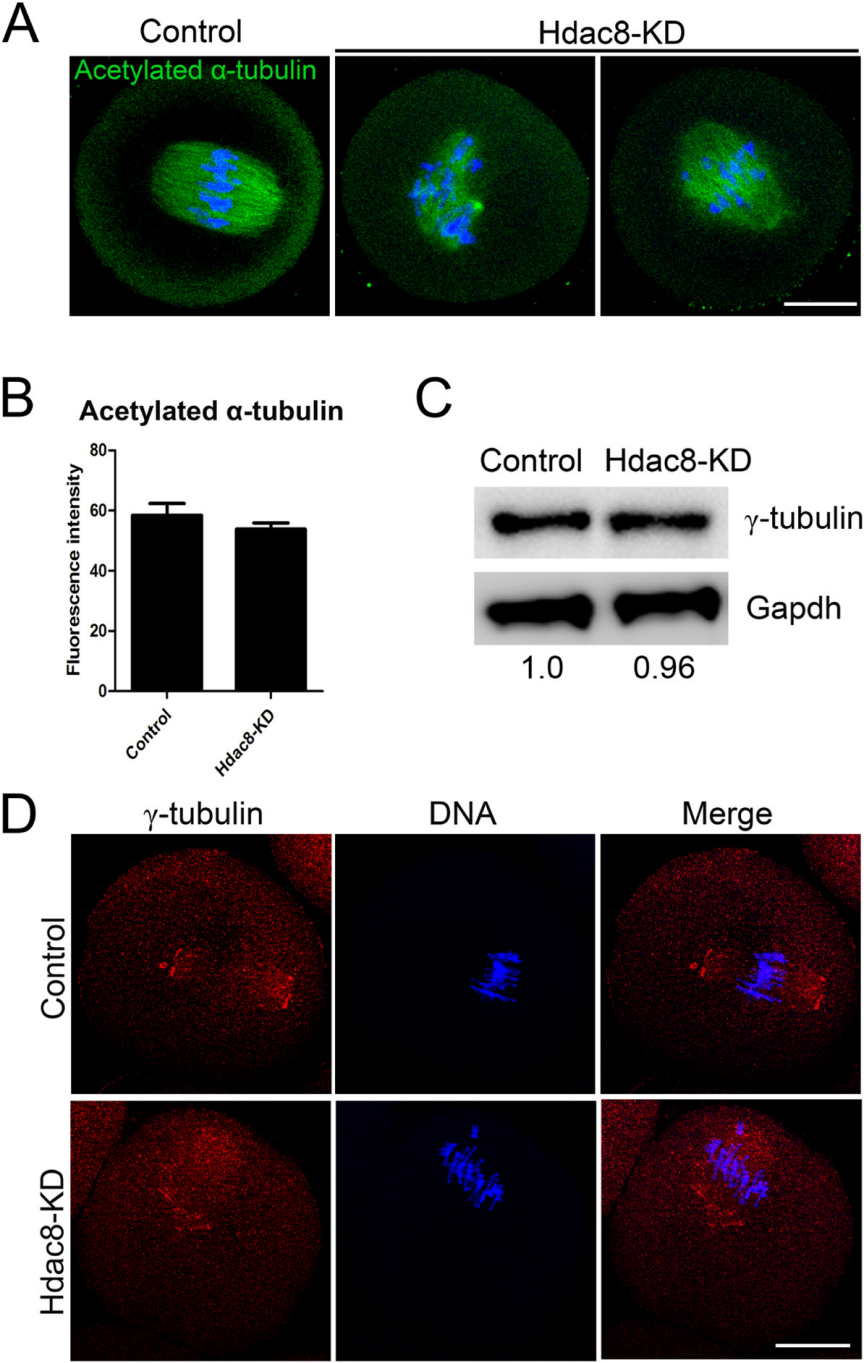
Depletion of HDAC8 disrupts the localization of γ-tubulin in mouse oocytes **A**. Representative images of acetylated α-tubulin in control and HDAC8-KD oocytes. Oocytes were immunostained with anti-acetylated α-tubulin antibody and counterstained with Hoechst to visualize chromosomes. Scale bar, 20 μm. **B**. The immunofluorescence intensity was recorded in control and HDAC8-KD oocytes. Data were presented as mean percentage (mean ± SEM) of at least three independent experiments. Asterisk denotes statistical difference at a *p < 0.05* level of significance. **C**. Protein levels of γ-tubulin in control and HDAC8-KD oocytes. The blots were probed with anti-γ-tubulin antibody and anti-Gapdh antibody, respectively. **D**. Localization of γ-tubulin in control and HDAC8-KD oocytes. Oocytes were immunostained with anti-γ-tubulin antibody and counterstained with Hoechst. Scale bar, 20 μm.

As HDAC8 plays critical roles in spindle assembly, we further investigated its possible participation in microtubule stability and organization. Thus we first detected the acetylation level of α-tubulin, a sign of stabilized microtubules, in the absence of HDAC8 in mouse oocytes. However, there were no significant differences of acetylated α-tubulin found between HDAC8-depleted oocytes and controls (53.82 ± 2.09, n=46 vs 58.43 ± 3.96%, n=42 control, *p* < 0.05; Figure 6A, 6B), implying that HDAC8 might not modulate the microtubule stability. Since we have observed the colocalization of HDAC8 with γ-tubulin, we then assessed the effect of HDAC8 depletion on γ-tubulin. The protein level of γ-tubulin did not show any changes in HDAC8-depleted oocytes when performing western blotting analysis (Figure 6C), but a large number of dislocated γ-tubulin was truly observed following HDAC8 depletion (Figure 6D), suggesting that γ-tubulin might be one of the downstream effector protein mediating the role of HDAC8 in microtubule organization and spindle assembly.

## DISCUSSION

HDACs typically induce histone deacetylation and repress gene transcription. However, recent studies suggest that some HDACs, such as HDAC6, are present in the cytoplasm of non-muscle cells, which have been implicated in regulating cell migration and microtubule dynamics [9]. Moreover, HDAC1 and HDAC2 play important roles in regulating kinetochore functions and chromosome segregation during oocyte meiosis and preimplantation embryo development [9]. HDAC8 is a zinc dependent class I histone deacetylase that plays a critical role in a wide aspects of biological processes, ranging from transcriptional regulation, sister chromatid separation, virus infection, energy homeostasis to smooth muscle contraction [18, 21, 26, 27]. Previous studies have shown that HDAC8 is ubiquitously expressed and localizes to the nucleus or the cytoplasm [12, 28] during mitosis. However, in the present study we observed, using immunofluorescent analysis coupled with confocal microscopy, that HDAC8 was concentrated on the spindle poles and colocalized with γ-tubulin during mouse oocyte meiotic maturation, suggesting that HDAC8 might have a function in spindle organization.

To test this hypothesis, we examined the spindle assembly following HDAC8-targeting siRNA microinjection. Expectedly, our data revealed that oocytes depleted of HDAC8 exhibited a significantly higher proportion of spindle organization failure and chromosome misalignment, indicating that HDAC8 could act as a regulatory part to function in normal spindle assembly in mouse oocytes. In addition, compromised K-M attachments were also observed in HDAC8-depleted oocytes, finally leading to the generation of aneuploid eggs.

Since HDAC8 is a deacetylase, another question is whether the involvement of HDAC8 in spindle assembly depends on its enzymatic activity. To test it, HDAC8-specific inhibitor PCI-34051 was used to inhibit the deacetylase activity. Consistent with a recent study [29, 30], the inhibitor did not alter the protein level of HDAC8. Notably, we found that inhibition of HDAC8 by PCI-34051 showed the similar phenotypes in spindle assembly, chromosome alignment and maintenance of euploidy to those resulting from RNAi silencing. Thus, these data illustrate that the deacetylase activity is required for the functions of HDAC8 during oocyte meiosis.

The regulation of HDAC8 in the spindle assembly further prompted us to determine its possible roles in the microtubule dynamics and organization. Tubulin acetylation, a post-translational modification that occurs on Lys-40 of the α-tubulin subunit [31, 32], is abundant in stable microtubules but is absent in dynamic subcellular structures [33]. It is well known that HDAC6 is a α-tubulin deacetylase that controls the microtubule stability in both mitosis and meiosis [34, 35]. To test if HDAC8 also has the deacetylase activity on the α-tubulin, we measured the fluorescent intensity of acetylated α-tubulin in the absence of HDAC8. The quantitative result did not show any difference of the signal intensity between control and HDAC8-depleted oocytes, indicating that microtubule stability is not affected by HDAC depletion. Given that HDAC8 is colocalized with γ-tubulin, we then wanted to examine if γ-tubulin would be the downstream effector of HDAC8. The result showed that although HDAC8 depletion did not alter the protein level of γ-tubulin, it indeed influenced the spindle pole localization of γ-tubulin, which implies that γ-tubulin might mediate the role of HDAC8 in microtubule organization and spindle formation. However, the underlying mechanisms regarding how HDAC8 regulates γ-tubulin need the future investigations.

Taken together, we provide several lines of evidence to demonstrate that HDAC8 exerts a critical function in spindle assembly and chromosome alignment to maintain the euploidy during mouse oocyte meiosis. Also, this role of HDAC8 requires its deacetylase activity and might be mediated by γ-tubulin.

## MATERIALS AND METHODS

### Antibodies

Rabbit polyclonal anti-HDAC8 antibody were purchased from Abcam (Cambridge, MA, USA; Cat#: ab187139); mouse monoclonal anti-α-tubulin-FITC antibody, mouse monoclonal anti-acetyl-α-tubulin (Lys-40) antibody and mouse monoclonal anti-γ-tubulin antibody were purchased from Sigma (St. Louis, MO, USA; Cat#: F2168, T7451 and T6557); human anti-centromere antibody was purchased from Antibodies Incorporated (Davis, CA, USA; Cat#: CA95617); FITC-conjugated goat anti-rabbit IgG (H + L), TRITC-conjugated goat anti-rabbit IgG (H + L) and FITC-conjugated goat anti-mouse IgG (H + L) were purchased from Zhongshan Golden Bridge Biotechnology Co., LTD (Beijing, China).

### Oocyte collection and culture

All experiments were approved by the Animal Care and Use Committee of Nanjing Agricultural University, China and were performed in accordance with institutional guidelines. Female ICR mice (4–6 weeks) were sacrificed by cervical dislocation after intraperitoneal injections of 5 IU pregnant mare serum gonadotropin (PMSG) for 46 h. Fully-grown oocytes arrested at prophase of meiosis I were collected from ovaries in M2 medium (Sigma, St. Louis, MO, USA). Only those immature oocytes displaying a germinal vesicle (GV) were cultured further in M16 medium (Sigma, St. Louis, MO, USA) under liquid paraffin oil at 37°C in an atmosphere of 5% CO2 incubator for *in vitro* maturation. At different time points after culture, oocytes were collected for subsequent analysis.

### SiRNA interference

Fully grown GV-intact oocytes were microinjected with 5–10 pl of non-targeting or HDAC8-targeting siRNA (Genepharma, Shanghai, China) in M2 medium containing 2.5 μM milrinone. The working concentration of siRNA was 25 uM. To facilitate the degradation of mRNA by siRNA, microinjected oocytes were arrested at GV stage in M16 medium containing 2.5 μM milrinone for 24 h, and then transferred to milrinone-free M16 medium to resume the meiosis for further experiments. HDAC8 siRNA sequences: 5′-GCCCUGCAUAAACAAAUGATTUCAUUUGUUUAUGCAGGGCTT-3′.

### Immunofluorescence and confocal microscopy

Oocytes were fixed in 4% paraformaldehyde in PBS (pH 7.4) for 30 minutes and permeabilized in 0.5% Triton-X-100 for 20 min at room temperature. Then, oocytes were blocked with 1% BSA-supplemented PBS for 1 h and incubated with anti-HDAC8 (1:50), anti-acetyl-α-tubulin (Lys-40) (1:100), anti-α-tubulin-FITC (1:300), or anti-centromere (1:200) antibodies at 4°C overnight. After washing four times (5 min each) in PBS containing 1% Tween 20 and 0.01% Triton-X 100, oocytes were incubated with an appropriate secondary antibody for 1 h at room temperature. After washing three times, oocytes were counterstained with Hoechst 33342 (10 μg/ml) for 10 min. Finally, oocytes were mounted on glass slides and observed under a confocal laser scanning microscope (Carl Zeiss 700).

### Western blotting

A pool of 400 oocytes was lysed in 4 × LDS sample buffer (ThermoFisher, Waltham, MA, USA) containing protease inhibitor, and then separated on 10% Bis-Tris precast gels and transferred onto PVDF membranes. The blots were blocked in TBST (Tris-buffred saline containing 0.1% Tween 20) containing 5% low fat dry milk for 1 h at room temperature and then incubated with anti-HDAC8 antibody (1:1000) or anti-γ-tubulin antibody overnight at 4°C. After three times of washes in TBST, the blots were incubated with 1:10,000 dilution of HRP (Horse Radish Peroxidase) conjugated secondary antibodies for 1 h at room temperature. Chemiluminescence was detected with ECL Plus Western Blotting Detection System (GE, Piscataway, NJ, USA) and protein bands were visualized by Tanon-3900. The blots were then stripped and reblotted with anti-Gapdh antibody (1:5000) for loading control.

### Chromosome spreading

Oocytes were exposed to Tyrode's buffer (pH 2.5) for about 30 s at 37°C to remove zona pellucidae. After recovery in M2 medium for 10 min, oocytes were fixed in a drop of 1% paraformaldehyde with 0.15% Triton X-100 on a glass slide. After air drying, chromosomes were counterstained with PI and examined under a laser scanning confocal microscope.

### Statistical analysis

All percentages from at least three repeated experiments were expressed as mean ± SEM, and the number of oocytes observed was labeled in parenthesesas (n). Data were analyzed by paired-samples t-test, which was provided by GraphPad Prism5 statistical software. The level of significance was accepted as *p < 0.05*.
